# BIIJ embracing social media

**DOI:** 10.2349/biij.6.1.e1

**Published:** 2010-01-01

**Authors:** NA Kadri, KH Ng

**Affiliations:** 1 Department of Biomedical Engineering, University of Malaya, Kuala Lumpur, Malaysia; 2 Department of Biomedical Imaging, University of Malaya, Kuala Lumpur, Malaysia

**Keywords:** Internet, journal publication, social media

Taking up the line of reasoning of our January 2009 Editorial, it is now even more evident that the Internet technology is progressing so very rapidly, that the landscape of electronic publishing will continue its own evolution as well. The world is witnessing a revolutionary change in the paradigm of scholarly publishing [1].

Having been for a short period of time the domain of the social thirst that typifies the development of teenagers, social networking has crystallised into the modern day juggernaut of marketing strategy [2]. Retail marketing in this sense has many lessons to teach the scientific community, not the least of which is that 69% of adults in the United States alone have an Internet access, 23% of which use a social networking site [3].Time spent on social networking sites has tripled over the past year [4].Clearly, there is a change in our traditional patterns of communication.

Rarely do 300 year old technologies endure within the scientific community, but the scientific journal style of peer review has permeated academia to become an entrenched matter of course; an institution of its very own. It has never been questioned, never been thoroughly scrutinised, and in this manner, peer review has subscribed to the humble journal in the face of significant production costs that are rarely recoverable by authors. Indeed, a contribution to an academic journal is an exercise in benevolences; a labour of love [5].

While many other industry-specific professionals retain their copyright through patents, deeds and other legal instruments and devices, digital technology finally allows academics to at last protect their intellectual property through secure and controlled electronic licensing. Publication and presentation have now transformed into a single process [5].

Social networking also has the boon of inclusion. Scientific publications are disseminated to a far broader sector of the community than that possible with the medium of a journal, and due to wide accessibility to the Internet, the next generation of students are no longer deprived of scientific advances for an extended period of time. Content is available immediately to this generation, whose familiarity with this technology extends from infancy [6,7].

BIIJ has taken steps to exploit these global improvements, and endeavours to provide the scientific community with a state-of-the-art implementation that embraces every possible technological development in social networking. In doing so BIIJ will provide the scientific community a platform to establish a presence in the global spectrum, and will facilitate a certain organisational framework within which information can be readily shared. No longer are dedicated members of the scientific community marginalised, ostracised and disconnected from their peers or the rest of the global community [8]. Social networking will propagate what has always been the objective of the scientific journal – to inspire reasoned and well substantiated proofs for the continuance of scientific discovery and the benefit of mankind.

As an active participant in the new frontier of scientific e-publication, BIIJ has invested in numerous complementary technologies that enhance the dynamic Web 2.0 paradigm of the Internet. Within this cyber infrastructure we have incorporated exciting new algorithms and methods to accelerate the versatility that folksonomy in particular has to offer [3]. Article-level commenting, RSS feeds, social networking on Twitter and Facebook, and dedicated academic visual networking are but a few of the novel implementations that BIIJ is now using to advance the cause of scientific learning.

## RSS

**Figure 1:**
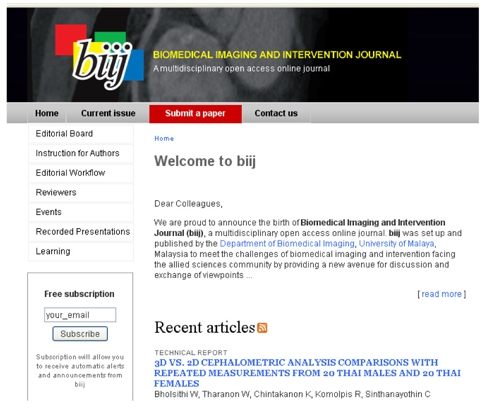
Screenshot of BIIJ homepage showing the RSS feed icon next to the ‘Recent articles’ heading.

**Figure 2:**
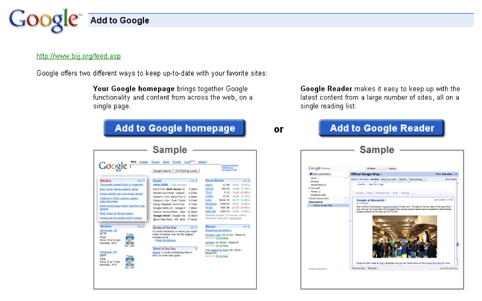
An option to use either Google™ Homepage or Google™ Reader when the RSS feed icon of BIIJ is clicked.

An acronym of Really Simple Syndication, RSS feeds provide the substance of material that has recently been amended or appended to a resource. An XML file allows uniform file interpretation by other applications that use an RSS reader (generic to contemporary web browsers) to incorporate such changes within their own operations. Perpetual vigilance is possible therefore, as updating is automated and requires no further assistance from the user. A mere click of the RSS icon on the BIJJ website (Fig. 1) will instigate the process, or alternatively users can paste the target URL into their browser’s RSS reader. Mozilla Firefox, for example, will prompt the user to choose between using the Google^TM^ Homepage or Google^TM^ Reader (Fig. 2), whenever a link containing a URL of an RSS feed is clicked.

Currently our RSS services provide identification of updates by title, author and a persistent link to the source material, but it is envisaged that soon an upgrade of our content management system will allow an abstract, and possibly the entire paper to be provided as part of the feed.

## TWITTER

This iconic innovation revolutionised social networking when it allowed users to send short messages to the Internet community at large, or to selected groups. Conversely, users could subscribe as followers of certain authors and receive messages exclusively from sources of their choice.

**Figure 3:**
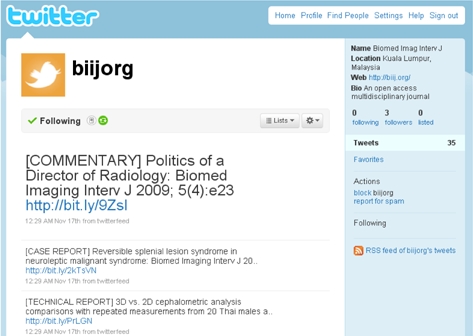
Screenshot of the Twitter page of BIIJ.

BIIJ utilises Twitter (available at http://twitter.com/biijorg) to inform followers of the latest news and journal updates, along with a link to the latest published papers and articles (Fig. 3). By incorporating a third party implementation known as Twitter Feed (http://twitterfeed.com), RSS technology is applied to Twitter, enabling update links to the journal to be automatically posted for all Twitter followers to receive.

## FACEBOOK

Originally spawned at Harvard University, Facebook allows users to maintain profiles and interact with friends online. Networks can be created to form associations of people with similar interests, and this social networking tool remains one of the most powerful online disseminators of profile information today.

BIIJ has its own Facebook profile (available at http://www.facebook.com/pages/Biomedical-Imaging-and-Intervention-Journal/188403815806), and allows ‘fans’ to communicate freely with one another, to keep abreast of the latest papers published, and also to submit their preferred links and resources, which in turn may be of interest to the general readers of the journal.

## SCIVEE

A funding initiative of the National Science Foundation (NSF), SciVee is a unique website that is dedicated to the scientific community and its objectives. Based at the University of California, it facilitates the uploading, viewing and sharing of scientific video, and uses Adobe’s Flash technology to display each clip. SciVee’s patent-pending proprietary application Rich Internet Applications (RIA), is then used to connect each video clip to a piece of scientific literature. A digital object identifier (DOI) is also assigned to each of the uploaded video clips, thus allowing a permanent link to be created.

The BIIJ page at SciVee (http://www.scivee.tv/user/biij) allows users to view recordings of lectures, presentations, and seminars online, or to download them directly from the SciVee site. The most viewed video currently is a presentation by Kwan-Hoong Ng at QAP in Mammography Workshop, Kuala Lumpur, held on 28-29 May 2009 [9].

## CONCLUSION

Through Web 2.0 applications that utilise dynamic keyword tagging and bookmarking to create tag clouds within a user profile, resources the world over are instantly at the fingertips of all those in the scientific community. Not only can authors share their academic findings with the world at large, but they can monitor related works and discoveries, engage in discussion of material, remain informed of funding criteria, availability and allocation, and through tag cloud analysis can examine the interests of colleagues. In so doing, the scientific community can share information about themselves and can engage in data mining techniques that will endure to be the most comprehensive yet achieved by science.

It remains imperative therefore for BIIJ to continually revise the delivery of scientific material to the global community, and ensure that it meets the standards of integrity and precision that is expected of the scientific fraternity. Along with the emergence of further technological advances in folksonomy and digital media, BIIJ will continue to bring coherence and unity to the collective body of scientific knowledge that is our legacy to the world. We will continue to monitor the impact of such social networking tools and to adapt to the changing landscape accordingly.
